# Gian Maria Fimia (1967–2024), an outstanding scientist with immense human qualities

**DOI:** 10.1038/s41419-024-06532-w

**Published:** 2024-03-04

**Authors:** Mauro Piacentini, Marco Tripodi, Gerry Melino, Giuseppe Ippolito

**Affiliations:** 1 Department of Biology, TOR, University of Rome “Tor Vergata”, Rome, Italy; 2 Department of Molecular Medicine, Sapienza University of Rome, Rome, Italy; 3 Department of Experimental Medicine, TOR, University of Rome “Tor Vergata”, Rome, Italy; 4 Chair of Infectious Diseases, Saint Camillus International University of Health Sciences, Rome, Italy

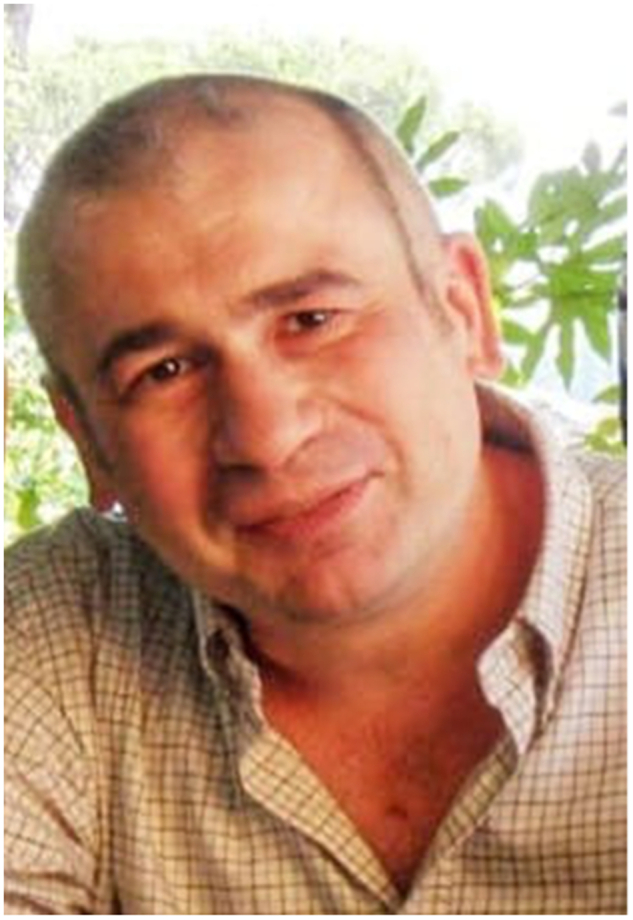

*“I am not afraid of death,” said Rita Levi-Montalcini. “If I die tomorrow or in a year, it is the same – it is the message you leave behind you that counts, and the young scientists who carry on your work.”*

With immense sadness, we write and honor Gian Maria who passed away in Rome aged only 56 years. Gian Maria (Giamma to his close friends) was a dear friend and an inspiration for his generation, not simply because of his professional achievements, but equally for his warm personality, exemplary hard playing life, and unbounded enthusiasm. His premature death, on January 22, 2024, following a tragic unexpected and extremely rapid illness, has left all his family and friends speechless.

Giamma started his career in 1992 at the University of Rome “La Sapienza” with a PhD under the guidance of Professor Paolo Amati. Following his Post-doc training at the Institute of Genetics, Molecular and Cellular Biology (IGBMC) in Strasbourg, France, in the laboratory of Prof. Paolo Sassone-Corsi (1996–2000), he then joined, in the year 2000, the Laboratory of Cellular Biology and Electron Microscopy at the National Institute for Infectious Diseases IRCCS “Lazzaro Spallanzani” in Rome where he worked until the end. In 2019, Gian Maria Fimia was appointed Full Professor of Applied Biology at the Department of Molecular Medicine, University of Rome “La Sapienza”. Between 2014 and 2017, he was also visiting professor at the Federal University of Sao Paulo in Brazil. Until now, he was an active member of the editorial board of our journal *Cell Death & Disease*.

Gian Maria Fimia was a world expert in cellular and molecular biology. His unique scientific qualities where already evident at the time of his work with Paolo Sassone Corsi where he dissected the regulation of gene expression of the CREB family transcription factors, which controls the expression of a large number of genes in response to distinct signaling pathways. These studies, resulting in the publication of pivotal *Nature* and *Science* articles, had a seminal impact in the understanding of the regulation of transcription in eukaryotes. The scientific achievement of Gian Maria Fimia continued to grow in Rome, at the Spallanzani Institute, with the discovery of Ambra1, a key player in the regulation of autophagy and cell cycle, also out in *Nature* in 2007. Gian Maria was also the first to show that the Cullins E3 ubiquitin ligases regulate the onset and termination of autophagy by dynamically interacting with Ambra1. Ironically, today Ambra1 finds its first clinical application in the prognosis of the progression of neoplasms, including melanoma. Needless to say, Gian Maria’s work has contributed in a fundamental way to the scientific reputation that Spallanzani enjoys today both nationally and internationally.

In addition to basic science, he worked on many translational aspects of research on infectious diseases, such as HIV, tuberculosis and viral hepatitis. His intent was on moving the advancements of scientific knowledge into potential application to diagnosis and treatment. Gian Maria approached all that he did with remarkable energy, optimism, and enthusiasm. He was one of the few to understand the rapid evolution of infectious disease research, and to wager on the possibility of establishment of research activities, based on the model of “Network Medicine” to perform innovative projects using various kinds of data collected on pathological phenotypes aimed at improving and qualifying the care response, with the final aim to build innovative disease management models.

He was a much appreciated member of funding committees, expert panels, society scientific boards and editorial roles for scientific journals. We think that the best way to remember Gian Maria is to follow his example as well as his ongoing projects, with the same passion and dedication that he demonstrated until his final days, despite his physical condition being so truly precarious. This is the last precious teaching, among many, that Gian Maria has given to us. We must treasure this message. He leaves a great void in the national and international scientific community.

Beside these scientific merits, we remember Giamma as a person who possessed a pronounced sense of justice and the unconditional will to find solutions that added value for all. Gian Maria was a person of rare mental honesty and sound moral principles which he constantly applied in his work and everyday life with great consistency, associating them with great professionalism. Very few people with such abilities and merits put themselves so little in the spotlight as he always did. Anyone who needed good advice was always in the right place with Giamma. It was important for him to support young colleagues at all times and to help them move forward on their scientific or clinical career path. No doubt his success as a leader of younger scientists was also due to his human approach and respect for others, and indeed the loss of his human qualities is what we will miss the most.

It has been a unique honor and a privilege to have Gian Maria Fimia as a colleague and a friend and we will miss him immensely. He will always live in our hearts. Our thoughts and deepest sympathy are with his wife Martina and daughter Francesca, his greatest loves of all.

